# Retroperitoneal abscess with subcutaneous extension: case report of a rare complication of percutaneous renal biopsy

**DOI:** 10.1186/s12882-018-1112-1

**Published:** 2018-11-09

**Authors:** Prashan Buddhika Illeperuma, Harsha Anuruddhika Dissanayake, Eranga Sanjeewa Wijewickrama

**Affiliations:** 1 University Medical Unit, National Hospital of Sri Lanka, Colombo, Sri Lanka; 2 Department of Clinical Medicine, Faculty of Medicine, University of Colombo, Colombo, Sri Lanka

**Keywords:** Renal biopsy, Perinephric abscess, Post-renal biopsy abscess

## Abstract

**Background:**

Infective complications following percutaneous renal biopsy are rare, even among immunocompromised. However it is important to be vigilant about such complications, to detect them early and prevent morbidity and mortality. We report a case of retroperitoneal abscess with extension to subcutaneous plane after a renal biopsy.

**Case presentation:**

A 42-year-old female with long standing cutaneous lupus underwent renal biopsy for evaluation of nephrotic range proteinuria. She was on high dose prednisolone complicated with steroid induced hyperglycaemia. Eight weeks after the biopsy she presented with left flank pain, malaise and fever. There was a tender subcutaneous induration over the biopsy site. Contrast CT abdomen showed a retroperitoneal abscess with subcutaneous extension along the path of the biopsy needle. This was successfully treated with surgical drainage and broad-spectrum antibiotics.

**Conclusions:**

Infections and abscess formation are rare but serious complications of renal biopsy. Immunocompromised state is a potential risk factor. Possible mechanisms and measures for prevention and early detection of this rare complication are discussed.

**Electronic supplementary material:**

The online version of this article (10.1186/s12882-018-1112-1) contains supplementary material, which is available to authorized users.

## Background

Infective complications following percutaneous renal biopsy are rare. Those are limited to case reports of perinephric abscesses, pyelonephritis and bacteraemia [1]. Many patients who undergo renal biopsy are often immunocompromised either due to underlying disease or immunomodulatory therapy. Despite this, infections in relation to renal biopsy have been exceedingly rare, even among kidney transplant recipients. We report a case of retroperitoneal abscess, which extended to subcutaneous tissues through muscles of the posterior abdominal wall, developed following a renal biopsy, a phenomenon not described before.

## Case presentation

A 42 year old female with cutaneous lupus for 16 years was evaluated for new onset hypertension and ankle oedema of 2 months duration. She was found to have a nephrotic range proteinuria (3.7 g per day) with microscopic haematuria and underwent renal biopsy for suspected lupus nephritis. She did not have coagulopathy, local skin sepsis or uncontrolled hypertension at the time of the biopsy. The procedure was performed under ultrasound guidance, adhering to aseptic precautions by an experienced specialty trainee in nephrology. Two cores were obtained with two passes using a Histo Automated Spring-loaded renal biopsy gun with a 16G needle. No complications were observed during the immediate post-procedure period. Patient did not develop undue pain, haematuria or overt bleeding from the biopsy site. She was discharged from hospital the next day.

She was on prednisolone 60 mg daily and had steroid induced diabetes mellitus. Her glycemic control was poor (HbA1c 9.0%, fasting plasma glucose 188 mg/dL) while being on treatment with metformin 750 mg thrice daily and gliclazide 40 mg twice daily.

Eight weeks later she was re-admitted with pain in the left flank, intermittent fever and malaise for 1 week. She did not have urinary symptoms, haematuria, nausea or vomiting.

Her past medical, surgical, gynaecological and family history was otherwise unremarkable. She was a housewife, leading an active lifestyle, well supported by family members and was well compliant with treatment.

On admission, she was ill, febrile (37.5 °C), had tachycardia (112 beats per minute) with normal blood pressure (120/70 mmHg), respiratory rate (18 per minute) and oxygen saturation (99% on ambient air). She was pale and had bilateral symmetrical pitting ankle oedema, malar rash, and erythematous desquamating rash over sun exposed areas. Abdominal examination revealed an exquisitely tender subcutaneous induration in the left flank without overlying erythema, warmth or rash. Cardiovascular, respiratory and neurological examinations were unremarkable.

Investigations revealed a neutrophil leukocytosis (total white cell count 22 300 / mm^3^, neutrophils 88% with left shift and toxic granules), elevated C-reactive protein (120 mg/L, reference < 6 mg/L) and erythrocyte sedimentation rate (88 mm 1st hour). She also had normochromic normocytic anaemia (haemoglobin 8.9 g/dL), normal renal functions (Creatinine 57 micmol/L) and normal liver biochemistry except for hypoalbuminaemia (26 g/L). Renal biopsy was reported as having insufficient tissue as it contained only tubules, without any glomeruli.

**Fig. 1:**
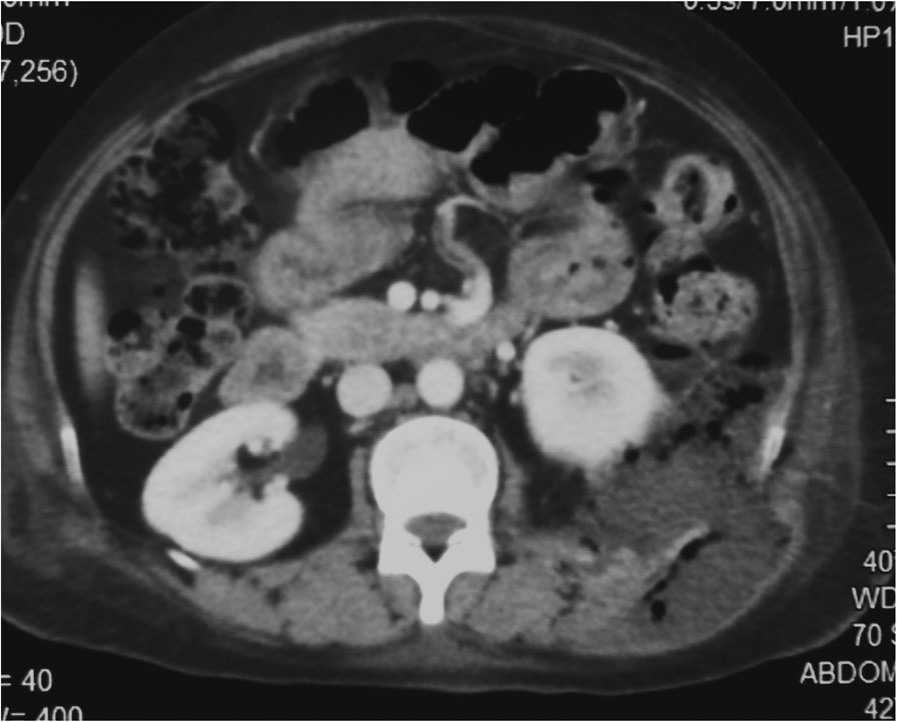
Contrast enhance CT abdomen showing a retroperitoneal abscess behind the left kidney which had extended to subcutaneous tissue plane through a defect in the posterior abdominal wall

Urinalysis showed proteinuria and microscopic haematuria without pyuria. Urine and blood cultures grew no organisms. Ultrasound scan of the abdomen showed a subcutaneous hypoechoeic area over the left flank suggestive of a fluid collection. A contrast enhanced CT scan of the abdomen was done which showed a retroperitoneal collection of pus that extended in to the subcutaneous tissues through the muscles of the posterior abdominal wall (Fig. 1). No communication was reported between the abscess and the renal tissue.

She underwent incision and drainage of the abscess, which drained 400 mL of blood stained pus. Collection was found to be extending from the retroperitoneal region to the subcutaneous tissue plane, two regions communicating through a channel that penetrated the posterior abdominal wall musculature. The abscess had no communication with renal tissues or the collecting system. Pus culture isolated an extended spectrum beta lactamase producing *Escherichia coli*. She was treated with intravenous meropenem 1 g 8 hourly along with regular debridement of the surgical site.

Her symptoms gradually resolved with treatment and inflammatory markers returned to normal. Follow up imaging with ultrasonography did not reveal any residual collection. Two weeks later, she was discharged from in patient care with a plan for repeat biopsy from the right kidney.

## Discussion & conclusions

Infective complications are rare after renal biopsy. These include pyelonephritis, perinephric abscesses and bacteraemia. In a follow up of 1000 patients who underwent renal biopsy, González-Michaca et al. [1] reported infective complications in only 3 patients with one having a perinephric abscess, one having pyelonephritis and the other one having bacteraemia. Thus the incidence of infective complications was only 0.3% in this series. Similarly a retrospective analysis of 1832 renal biopsies over 37 years reported infections to affect only 0.2% of the biopsied patients [2]. Other prospective and retrospective data from databases have shown similar low incidence of perinephric abscesses (0.3%) [3]. In contrast, bacteraemia was shown to be commoner when renal biopsy was performed on those with pyelonephritis, affecting 6.2% of such patients [4]. However, most such cases were reported at least 20 years ago. More recent data from large cohorts of patients report no infective complications in relation to renal biopsy [5, 6].

There are several mechanisms by which an infection could develop following a renal biopsy. During the procedure, the biopsy needle may introduce skin commensals in to the renal parenchyma or surrounding connective tissues or muscles, which have relatively weak immune system. Similarly the needle may introduce skin commensals directly in to the blood stream through which the organisms may disseminate causing bacteraemia. Alternatively, path of the biopsy needle through soft tissues may leave a pathway along which the skin commensals may migrate in to the immune deprived connective tissues. Occult haemorrhage in to perinephric tissues following the biopsy may serve as a culture medium for the microorganisms to proliferate and disseminate. Occult perinephric haematoma is a common complication following renal biopsy detected on post procedure CT scans in 57–85% of those who undergo renal biopsies [7]. However infection of these haematomas are exceedingly rare [6]. Inadvertent injury to adjacent intestinal loops allowing entry of gut commensals to peritoneal cavity leading to peritonitis is a theoretical possibility, but has never been reported. Patients who undergo renal biopsy are also likely to have systemic disease with immune dysfunction due to the disease itself or as a result of immunosuppressive therapy making them more prone to infective complications.

Despite the multitude of predisposing mechanisms, infective complications following renal biopsies have been exceedingly rare in clinical practice. Strict adherence to aseptic precautions may at least partly contribute to the low incidence. Although the needle would introduce organisms to the connective tissues, relatively low perfusion and therefore anaerobic environment and low supply of nutrients would not facilitate survival and replication of organisms. Rapid epithelialization of skin puncture site following biopsy will prevent continued entry of organisms in to deeper tissue planes. Epithelialization following a clean surgical wound requires no immune response and depends on integrity of the epidermis and is therefore remains unaffected by immune dysfunction. Even in kidney transplant recipients who undergo graft biopsies, infections are exceedingly rare, despite being on immunosuppressants. Use of ultrasound guidance has minimized hemorrhage and inadvertent visceral injury thus minimizing infective complications.

It is very likely that our patient developed the retroperitoneal abscess as a complication of the renal biopsy. The abscess was anatomically localized to the retroperitoneal region adjacent to the biopsied kidney. It has tracked to the subcutaneous plane across the muscles and surrounding fascia. Infections tend to track along tissue planes and it is exceedingly rare for the infections to penetrate dense fascia and muscular tissues. It is likely that the infection tracked along the path of the needle in to the subcutaneous tissue plane. However, pus collection did not spread in to renal tissues. Kidney appeared unaffected radiologically and pus collection was separated from the kidney by intact Gerota’s fascia. Furthermore, urinalysis revealed no pyuria to suggest extension of infection in to collecting system. Inadvertent visceral injury contributing to the abscess was unlikely considering that the pus collection was entirely retroperitoneal. Furthermore, a visceral perforation would have caused peritonitis and sepsis within a few days of the procedure, which did not occur in the patient.

However, the organism isolated from the purulent drainage was an ESBL producing coliform, an organism unlikely to colonize the skin of a community dwelling otherwise healthy person [8]. We postulate that the cutaneous lupus altered the integrity of the skin as a barrier against infection resulting in it being colonized by this unusual organism during her recent hospitalization for the renal biopsy. In fact, a recent study has demonstrated that hospitalized patients have skin colonization with enterobacteriaceae [9]. Alternatively, the organism could have reached the site of infection through an occult renal infection or colonization that was present at the time of biopsy. Biopsy needle would have introduced the organisms to the soft tissue layer during the procedure.

Immune dysfunction secondary to lupus flare, high dose of prednisolone and the resultant deterioration in glycaemic control would have facilitated the development of the retroperitoneal abscess.

We suggest that strict adherence to aseptic precautions, use of correct technique to minimize multiple punctures and to avoid visceral injury as potential strategies to minimize infective complications following renal biopsy. Given the rarity of such complications it is unlikely that peri-procedure antibiotic prophylaxis would be cost effective. It is also important to be aware of this rare complication and to maintain a high index of suspicion in evaluating patients with suggestive clinical features with supportive radiological imaging and treat with appropriate antibiotics and surgical drainage.

In conclusion, this case illustrates the presentation, diagnostic approach and management of a rare infective complication following percutaneous renal biopsy. Dysfunction of the skin as a barrier to infections due to cutaneous lupus, colonization of the skin with drug resistant virulent organism during hospital stay, introduction of organisms to immune deprived connective tissues along the biopsy needle path, and tracking of the infection from retroperitoneal space to subcutaneous tissue plane along the path created by the biopsy needle and systemic immune deficient state secondary to lupus flare, immunosuppressive therapy and poorly controlled diabetes are the potential mechanisms by which our patient developed this complication. We emphasize the need for strict adherence to aseptic precautions and prompt management of immune deficient states such as diabetes as measures to prevent such complications. Patients as well as health care providers should be aware of this complication and its presentation and maintain a high index of suspicion to appropriately investigate and treat in order to prevent serious sequalae of sepsis (Additional file 1).

## Additional file

Additional file 1: Time line of disease evolution. (DOCX 36 kb)
