# Supplementary material for: Retroperitoneal abscess with subcutaneous extension: case report of a rare complication of percutaneous renal biopsy
Source: BMC Nephrol. 2018 Nov 9;19:319. doi: 10.1186/s12882-018-1112-1 (PMC6230226; doi:10.1186/s12882-018-1112-1)
Supplement: Supplementary file 1 — Time line of disease evolution. (DOCX 36 kb) [file 12882_2018_1112_MOESM1_ESM.docx]

42 year old female

Cutaneous lupus erythematosus since 2002, relapsed in 2017 December – on prednisolone 1mg/kg since then

Steroid induced diabetes: on metformin 750 mg tds and gliclazide 40 mg twice a day

Neutrophil leukocytosis, high CRP and ESR

UFR, urine culture, blood culture – no growth

Contrast CT abdomen – retroperitoneal abscess with subcutaneous extension

Tender subcutaneous induration over left flank

Admitted with worsening left flank pain, fever, malaise

Renal biopsy

Uncomplicated procedure and immediate post procedure period

Hypertension, nephrotic range proteinuria with bland sediment, normal renal functions

**November 2017**

**3.1.2018**

**23. 2.2018**

Left flank pain, low grade intermittent fever

Meropenem 1g 8 hourly IV commenced

**02.03.2018**

**03.03.2018**

Surgical drainage

**04.03.2018**

Pus culture – Escherichia coli, ESBL producer, sensitive to meropenem.

Gradual resolution of fever, pain, leukocytosis and inflammatory markers.

Regular repeated

wound debridement

**19.03.2018**

Follow up ultrasound – no focal collection

Discharged from in-ward care

**Conclusion:**

**Retroperitoneal abscess following renal biopsy, probably secondary to a hospital acquired infection. Abscess tracked along the path created by biopsy needle, in to subcutaneous place**
